# Preoperative activation of the renin–angiotensin system and myocardial injury in noncardiac surgery: exploratory mechanistic analysis of the SPACE randomised controlled trial

**DOI:** 10.1016/j.bja.2024.10.040

**Published:** 2024-12-20

**Authors:** Ana Gutierrez del Arroyo, Tom E.F. Abbott, Akshaykumar Patel, Salma Begum, Priyanthi Dias, David Brealey, Rupert M. Pearse, Vikas Kapil, Gareth L. Ackland, for the SPACE trial investigators

**Affiliations:** 1 Translational Medicine and Therapeutics, William Harvey Research Institute, Queen Mary University of London, London, UK; 2 Bloomsbury Institute of Intensive Care Medicine, University College London, London, UK; 3 UCL Hospitals NHS Foundation Trust, London, UK; 4 NIHR University College London Hospitals Biomedical Research Centre, London, UK; 5 Clinical Pharmacology & Therapeutics, William Harvey Research Institute, Queen Mary University of London, London, UK

**Keywords:** major cardiac events, myocardial injury, noncardiac surgery, perioperative care, renin–angiotensin-aldosterone system, *renin-angiotensin system* inhibitors

## Abstract

**Background:**

Hypertension therapy in older adults is often suboptimal, in part because of inadequate suppression of the renin–angiotensin–aldosterone system (RAAS). We hypothesised that distinct endotypes of RAAS activation before noncardiac surgery are associated with increased risk of myocardial injury.

**Methods:**

This was a prespecified exploratory analysis of a multicentre randomised controlled trial (ISRCTN17251494) which randomised patients ≥60 yr old undergoing elective noncardiac surgery to either continue or stop RAAS inhibitors (determined by pharmacokinetic profiles). Unsupervised hierarchical cluster analysis identified distinct groups of patients with similar RAAS activation from samples obtained before induction of anaesthesia, quantified by enzyme-linked immunoassays for plasma renin, aldosterone, angiotensin-converting enzyme 2, and dipeptidyl peptidase-3. The primary outcome, masked to investigators and participants, was myocardial injury (plasma high-sensitivity troponin-T).

**Results:**

We identified three clusters, with similar proportions of RAAS inhibitors randomised to stop or continue. Cluster 1 (*n*=52; mean age [standard deviation], 75 yr [8 yr]; 54% female) and cluster 3 (*n*=25; 75 yr [6 yr]; 44% female) had higher rates of myocardial injury (23/52 [44%] and 13/25 [52%], respectively), compared with cluster 2 with 51/164 (31.1%; *n*=153; 70 yr [6] yr; 46% female; odds ratio: 1.95, 95% confidence interval (CI) 1.12–3.39, *P*=0.018). Cluster 2 was characterised by lower N-terminal pro-B-type natriuretic peptide (NT-proBNP) concentration (mean difference 698 pg ml^−1^, 95% CI 576–820 pg ml^−1^) and higher renin concentration (mean difference 350 pg ml^−1^, 95% CI 123–577 pg ml^−1^), compared with clusters 1 and 3 which had higher rates of myocardial injury.

**Conclusions:**

This mechanistic exploratory analysis suggests that effective preoperative RAAS inhibition is associated with lower risk of myocardial injury after noncardiac surgery, independent of stopping or continuing RAAS inhibitors before surgery.

**Clinical trial registration:**

ISRCTN17251494.

**Editor's key points**

• Surgical patients most are commonly prescribed renin–angiotensin system inhibitors (RASi) for cardiometabolic disease and hypertension, but treatment is often suboptimal.
• In this prespecified mechanistic exploratory study of the SPACE trial, the authors hypothesised that endotypes of renin–angiotensin–aldosterone system activation predispose patients to postoperative myocardial injury independent of RASi cessation or continuation.
• Effective preoperative RAAS inhibition was associated with lower risk of myocardial injury after noncardiac surgery independent of stopping or continuing RAAS inhibitors before surgery.
• Further work to explore whether continuation or preoperative intensification of RASi therapy might help reduce perioperative myocardial injury is warranted.

Despite advances in perioperative care, postoperative complications still lead to delayed recovery and accelerated mortality even after discharge from hospital.^1^^,^^2^ The complexity of perioperative pathophysiology necessitates, in part, a patient-specific approach to understand perioperative pathophysiology in more depth, thereby identifying opportunities to reduce the burden of postoperative complications.^3^

Surgical patients most at risk of sustaining myocardial injury and subsequent complications^4^ after noncardiac surgery^5^^,^^6^ are commonly prescribed renin–angiotensin system inhibitors (RASi) for cardiometabolic disease and hypertension.^7^^,^^8^ Hypertension therapy in older adults is often suboptimal,^9^ which can impact on cardiac complications after noncardiac surgery by failing to reduce activation of the renin–angiotensin system and inflammation.^10^ The noncanonical axis of the renin–angiotensin system counteracts the deleterious effects of angiotensin II (ANG-II), largely through angiotensin-converting enzyme-2 (ACE2).^11^ As a homologue of ACE, ACE2 converts ANG-II into the organ-protective mediator ang1-7. Dipeptidyl peptidase-3 (DPP-3) also positively regulates the renin–angiotensin–aldosterone system (RAAS) pathway by degrading circulating ANG-II.^11^ Accordingly, inadequate treatment with RASi can predispose noncardiac surgical patients to organ injury by failing to reduce aldosterone through the negative feedback effect of ANG-II on renin release or maintaining cellular protection via the counter-regulatory RAAS.

In this prespecified, mechanistic exploratory study of the SPACE multicentre, randomised trial of stopping or continuing RASis according to their individual pharmacokinetic profile before noncardiac surgery,^12^ we hypothesised that endotypes of RAAS activation predispose patients to myocardial injury within 48 h of surgery independently of RASi cessation or continuation.

## Methods

### Study design and participants

This randomised controlled trial was conducted in accordance with International Council for Harmonisation (ICH) Good Clinical Practice Guidelines and applicable laws and regulations. The study protocol was approved by London research ethics committee (16/LO/1495) and the Medicines and Healthcare products Regulatory Agency (UK). Each participant provided written informed consent. The SPACE trial was reported in accordance with CONSORT guidelines and registered publicly (ISRCTN17251494). From July 31, 2017 to October 1, 2021, the Stopping Perioperative ACE inhibitors/ARBs (SPACE) phase 2a trial (EudraCT:2016-004141-90) randomised adults aged ≥60 yr to either continue or stop prescribed RASi according to the individual pharmacokinetics of each drug. Adults prescribed RASi aged ≥60 yr, with American Society of Anesthesiologists physical status 3 or above undergoing elective major surgery requiring general anaesthesia lasting >120 min were eligible. Exclusion criteria included current participation in any other interventional clinical trials and myocardial infarction within the 3 months preceding surgery. We did not exclude patients receiving angiotensin-converting enzyme inhibitor (ACEi) or angiotensin receptor blocker (ARB) therapy for left ventricular dysfunction (ejection fraction [EF] <50%).

### Randomisation and masking

Randomisation was performed centrally, with minimisation by centre, RASi, and type of surgery. After randomisation, participants received confirmation of which treatment group they have been allocated to and reminded of their randomised allocation by daily telephone call, text message, or both, or in person if they were in hospital. All laboratory personnel undertaking troponin measurements and ELISAs were masked to study details and trial allocation.

### Perioperative drug management

RASi were restarted after surgery on the morning of postoperative Day 2 in accordance with recommendations of the European Society of Cardiology (ESC) guidelines.^13^ RASi were recommenced at the same dose as that prescribed before surgery. Resumption of RASi therapy was delayed on postoperative Day 2 if systolic blood pressure was <90 mm Hg in the preceding 12 h, vasoactive therapy was required to maintain blood pressure, or with evidence of acute kidney injury.^14^ Usual perioperative practice was delivered in accordance with recent ESC perioperative guidelines.^13^

### Primary and secondary endpoints

The primary endpoint was myocardial injury, defined by troponin-T levels (Roche, Basel, Switzerland) using the VISION study thresholds.^1^ As recommended by the Standardized Endpoints Consensus guidelines for cardiovascular outcomes in perioperative care,^15^ major adverse cardiovascular events and length of hospital stay are also reported.

### Explanatory variables

Blood samples collected before the induction of anaesthesia and on the morning 24 h and 48 h after surgery. Batched analyses of plasma samples stored at −80ºC were undertaken for troponin (Doctor's Laboratory, London, UK) and RAAS components. From samples obtained on the day of surgery before induction of anaesthesia, we quantified renin (BMS2271, Invitrogen, Carlsbad, CA, USA), aldosterone (ADI-900-173, Enzo, Farmingdale, NY, USA) ACE2 (DY33-05, R+D Systems, Abingdon, UK), DPP-3 (E3112Hu, Bioassay Technology Laboratory, Shanghai, China), and N-terminal pro-B-type natriuretic peptide (NT-proBNP) (ab263877, Abcam, Cambridge, UK) using established ELISAs performed by personnel masked to study details (Infinite 200 Pro plate reader, Tecan, Männedorf, Switzerland). Standard curves for each ELISA exceeded *R*^2^ values ≥0.99 (Supplementary material).

### Statistical analysis

A statistical analysis plan was published before biochemical analyses were undertaken (https://www.qmul.ac.uk/ccpmg/sops--saps/statistical-analysis-plans-saps). We used cluster analysis to identify RAAS endotypes, a data reduction technique designed to uncover subgroups of observations within a dataset. A cluster is defined as a group of observations that are more similar to each other than they are to the observations in other groups. This has been used in cardiovascular medicine to identify heterogeneity in pathophysiological states including heart failure,^16^ left ventricular dysfunction,^17^ and echocardiographic phenotyping.^18^ Optimal cluster number was determined using NbClust (R4.3.2; Supplementary material).^19^ We used unsupervised hierarchical cluster analysis to generate homogeneous groups of participants by reducing the preoperative plasma values for renin, aldosterone, ACE2, DPP-3, and NT-proBNP. Primary and secondary outcomes were analysed by Fisher's exact test and log-rank test, as appropriate. Maximal troponin and RAAS mediator concentrations were analysed by one-way analysis of variance (anova), with *post hoc* Tukey–Kramer testing. Analyses were performed using NCSS 2023 (Kaysville, UT, USA).

## Results

### Cluster characteristics

**Fig 1:**
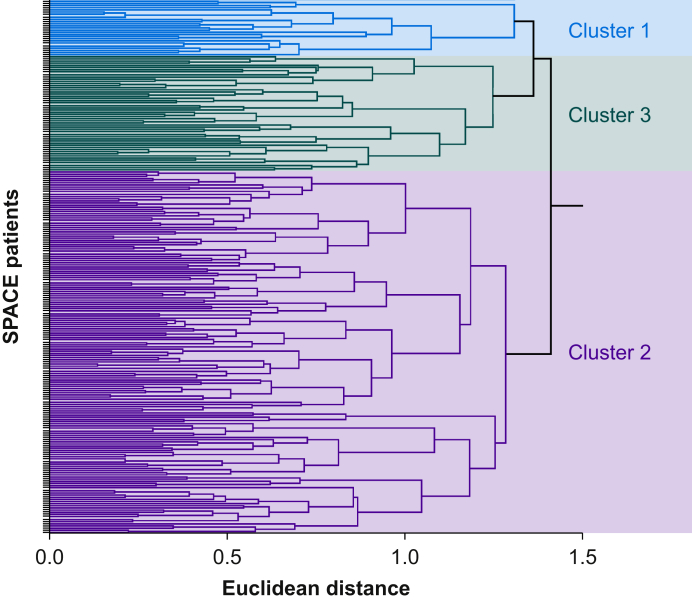
Unsupervised hierarchical cluster analysis. Clustering of preoperative renin–angiotensin–aldosterone system (RAAS) characteristics, determined by unsupervised hierarchical cluster analysis after determination of optimal cluster number by NBClust (Supplementary material).

**Fig 2:**
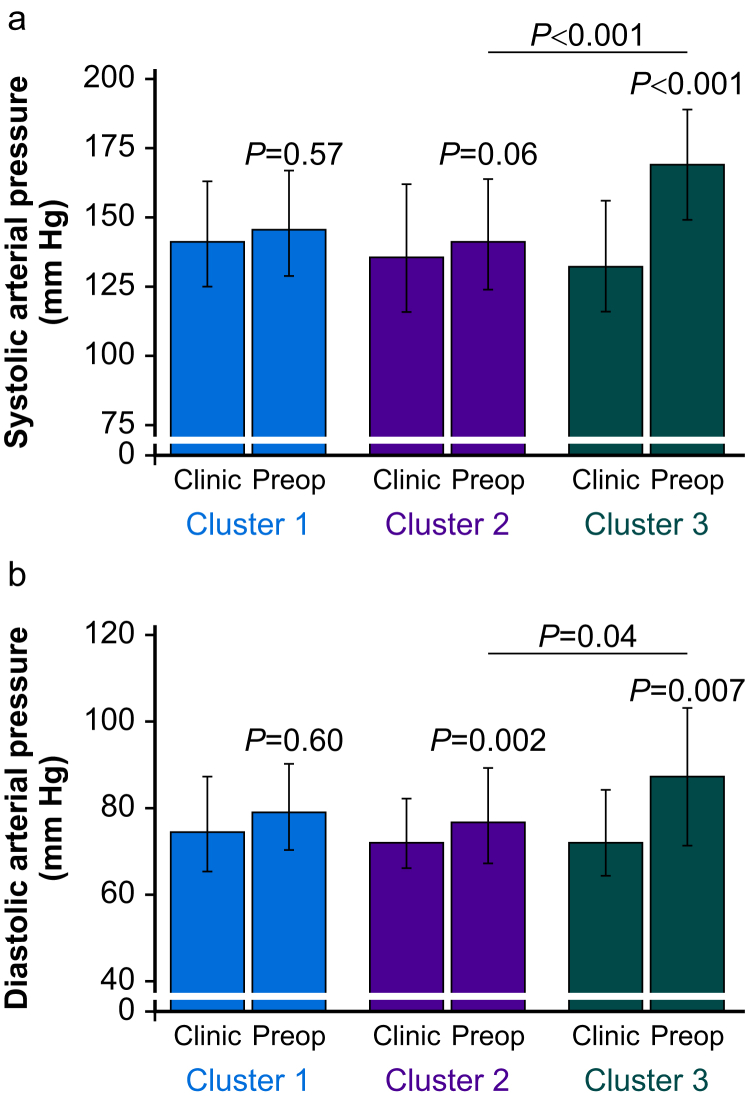
Arterial pressure changes before and after randomisation in each cluster. (a) Systolic arterial blood pressure in preoperative clinic and on the day of surgery for each cluster identified by unsupervised cluster analysis. (b) Diastolic arterial blood pressure in the preoperative clinic and on the day of surgery for each cluster identified by unsupervised cluster analysis. *P*-values refer to within-group *post hoc* Tukey–Kramer comparisons, plus comparison between clusters 2 and 3.

Cluster analysis distinguished three groups of participants by biochemical characteristics of RAAS activation before surgery (Fig. 1). Participants within each cluster underwent similar types of surgery and were prescribed similar proportions of ACEis and ARBs before surgery (Table 1). A preoperative diagnosis of coronary artery disease was present to a similar extent across each cluster (χ^2^=0.22; *P*=0.90). The proportion of RASi stopped or continued before surgery by random allocation were also similar, with 24/52 (46%) in cluster 1, 84/164 (51%) in cluster 2, and 12/25 (48%) in cluster 3 (χ^2^=0.44; *P*=0.80). Cluster 3, which received more antihypertensive medications, also developed 34 mm Hg (95% confidence interval [CI] 15–53 mm Hg) higher systolic, and 14 mm Hg (95% CI 4–24 mm Hg) higher diastolic blood pressure after randomisation compared with clusters 1 and 2 (Fig. 2).

**Table 1:** Participant characteristics by cluster. Data are *n* (%), mean (standard deviation) or median (interquartile range). ACEi, angiotensin-converting enzyme inhibitor; ARB, angiotensin receptor blocker; COPD, chronic obstructive pulmonary disease; GFR, glomerular filtration rate; RASi, renin–angiotensin system inhibitor.

	Cluster 1	Cluster 2	Cluster 3
*n*	52	164	25
Female, *n* (%)	28 (54)	75 (46)	11 (44)
Age (yr)	75 (8)	70 (6)	75 (6)
Haemoglobin before surgery (g L^−1^)	127 (24)	134 (14)	128 (30)
Estimated GFR (ml min^−1^ 1.73 m^−2^)	91 (32)	87 (29)	101 (56)
ACEi, *n* (%)	33 (63)	101 (62)	13 (52)
ARBs, *n* (%)	19 (37)	63 (38)	12 (48)
Randomised to stop ACEi/ARB, *n* (%)	24 (46)	84 (51)	12 (48)
Extra-abdominal surgery	42 (81)	134 (82)	18 (72)
Coronary artery disease	12 (23.1)	33 (20.1)	5 (20)
COPD, *n* (%)	3 (6)	26 (16)	0
Asthma, *n* (%)	6 (12)	20 (12)	2 (8)
Diabetes mellitus, *n* (%)	17 (33)	50 (30)	6 (24)
Heart failure, *n* (%)	4 (8)	11 (7)	1 (4)
Cancer	15 (29)	38 (23)	8 (32)
Stroke, *n* (%)	2 (4)	13 (8)	3 (12)
Peripheral vascular disease	2 (4)	11 (7)	2 (8)
Hypertension, *n* (%)	51 (98)	160 (98)	23 (92)
Smoker, *n* (%)	2 (4)	16 (10)	2 (8)
Number of antihypertensives (1:2:3:4)	14:20:13:5	50:75:31:8	5:9:10:1
≥3 antihypertensives including RASi, *n* (%)	13 (25)	39 (24)	11 (44)
Beta blocker, *n* (%)	21 (40)	42 (26)	10 (40)
Calcium channel blocker, *n* (%)	18 (35)	60 (37)	7 (28)
Doxazosin, *n* (%)	6 (12)	13 (8)	4 (16)
Diuretic, *n* (%)	16 (31)	46 (28)	11 (44)
Statin, *n* (%)	33 (63)	122 (74)	16 (64)
Nitrate, *n* (%)	8 (15)	14 (9)	3 (12)
Antiplatelet drug	18 (35)	62 (38)	8 (32)

### Primary outcome: myocardial injury after surgery

**Fig 3:**
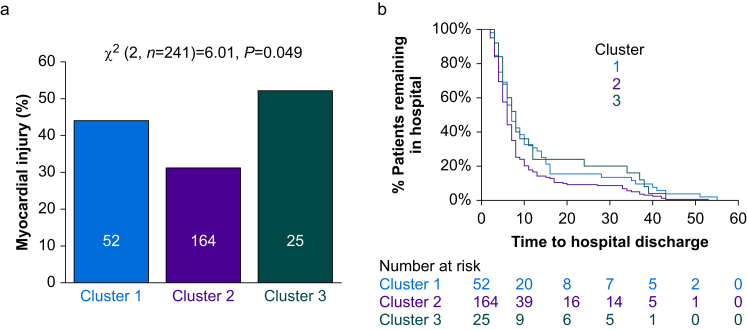
Primary outcome: myocardial injury. (a) Incidence of myocardial injury in each cluster. Numbers of participants per cluster are shown within each bar. (b) Length of hospital stay illustrated by time to hospital discharge in Kaplan–Meier plot for each cluster. Participants in cluster 2 had a shorter hospital stay (hazard ratio, 0.73; 95% confidence interval 0.56–0.95); *P*=0.012 by log-rank test).

For the primary outcome, clusters 1 and 3 had higher rates of myocardial injury (36/77 [47%] patients) with elevated troponin after surgery (Fig. 3a), compared with 51/164 (31.1%) patients in cluster 2 (odds ratio, 1.95; 95% CI 1.12–3.39; *P*=0.018). A sensitivity analysis excluding beta-blockers (which alter the aldosterone:renin ratio)^20^ showed similar results (odds ratio, 2.09; 95% CI 1.04–4.21; *P*=0.038, Supplementary material).

### Secondary outcomes

Mean peak troponin levels were 5 ng L^−1^ lower (95% CI 1–9 ng L^−1^) in cluster 2 patients, compared with clusters 1 and 3 (*P*<0.001). For the entire cohort, myocardial injury was associated with a prolonged hospital stay of 3 days (95% CI 1–5 days). Patients in cluster 2 had a shorter hospital stay (hazard ratio, 0.73; 95% CI 0.56–0.95; *P*=0.012, by log-rank test; Fig. 3b). Clinically defined cardiovascular complications were similar across each cluster (Table 1).

### Renin–angiotensin–aldosterone system characteristics of each cluster

**Fig 4:**
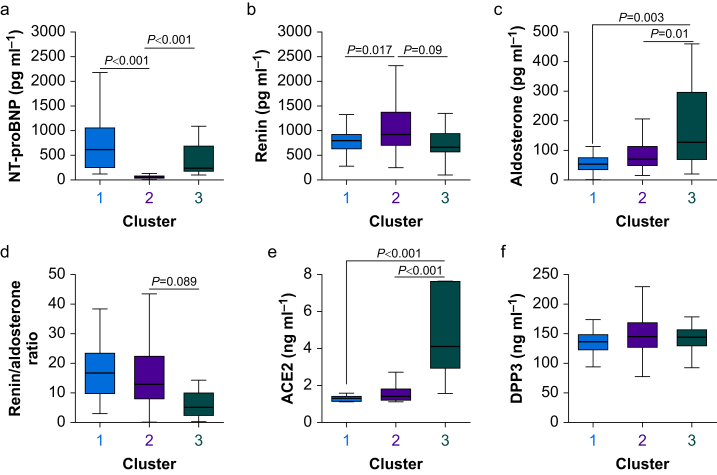
RAS endotypes: plasma levels of RAAS mediators. (a) NT-proBNP. (b) Renin. (c) Aldosterone. (d) Renin:aldosterone ratio. (e) ACE2. (f) DPP-3. All data are median (interquartile range). *P*-values refer to between-group comparisons, as derived from *post hoc* Tukey–Kramer comparisons. ACE2, angiotensin-converting enzyme-2; DPP-3, dipeptidyl peptidase-3; NT-proBNP, N-terminal pro-B-type natriuretic peptide; RAAS, renin–angiotensin–aldosterone system; RAS, renin–angiotensin system.

Cluster 2, with the lowest rate of myocardial injury, was characterised by lower NT-proBNP (mean difference 698 pg ml^−1^; 95% CI 576–820 pg ml^−1^; Fig. 4a) and higher renin (mean difference 350 pg ml^−1^; 95% CI 123–577 pg ml^−1^; Fig. 4b), compared with clusters 1 and 3 that had higher rates of myocardial injury. Cluster 3 had higher plasma aldosterone (mean difference 196 pg ml^−1^; 95% CI 30–362 pg ml^−1^; Fig. 4c and d) and ACE2 (mean difference 3.8 ng ml^−1^, 95% CI 1.0–6.6 ng ml^−1^) compared with clusters 1 and 2 (Fig. 4e). DPP-3 values were similar across each cluster (Fig. 4f).

### *Post hoc* analyses

After peer review, a *post hoc* examination of the relationship between absolute levels of troponin after surgery and preoperative RAAS components (also taking into account age, sex, and study allocation) was undertaken. We found that raised preoperative NT-proBNP (at a prognostic threshold >100 ng ml^−1^) and renin were strongly associated with sustaining myocardial injury (Supplementary material).

## Discussion

**Fig 5:**
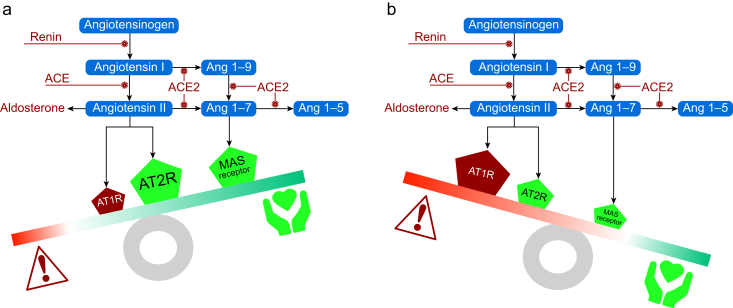
Preoperative RAS endotypes and factors altering risk of myocardial injury. (a) Effective ACE inhibition and angiotensin II blockade promote cardioprotective signalling pathways via reduction in ROS and inflammation. (b) Lack of effective RAAS suppression leads to excess aldosterone and ACE2 expression, promoting diastolic and endothelial dysfunction and worse perioperative outcomes. ACE2, angiotensin-converting enzyme-2; AT1R, Angiotensin II receptor type 1; AT2R, Angiotensin II receptor type 2; MAS, MAS receptor; RAAS, renin–angiotensin–aldosterone system; RAS, renin–angiotensin system; ROS, reactive oxygen species.

This exploratory predefined mechanistic substudy of the SPACE RCT found that participants with a preoperative RAAS endotype indicative of adequate RAAS inhibition or cardiovascular therapy had a lower risk of sustaining myocardial injury after noncardiac surgery. These findings were independent of whether the patients were randomised to continue or stop ACEis or ARBs in the SPACE RCT. Patients at lowest risk of sustaining myocardial injury had substantially lower preoperative NT-proBNP levels. Taken together, these data indicate that chronic undertreated RAAS activation contributes to perioperative myocardial injury (Fig. 5), and is consistent with the findings of the main SPACE trial where stopping ACEi/ARBs was associated with higher rates of myocardial injury.

The two clusters with higher rates of myocardial injury were characterised by distinct clinical features and biochemical indicators of RAAS activation that are mechanistically linked to cardiomyocyte damage. Cluster 3, comprising ∼10% patients, had marked elevation of plasma aldosterone, a key mediator in the pathogenesis of heart failure consistent with primary aldosteronism evident in 8–13% of nonselected patients with essential hypertension.^21^ Aldosterone increases cardiac inflammation, fibrosis, and myocardial hypertrophy through numerous mechanisms.^22^ Aldosterone impairs endothelial function by reducing nitric oxide production in endothelial cells through the inhibition of endothelial nitric oxide synthase activity.^23^ Aldosterone reduces glucose-6-phosphate dehydrogenase in endothelial cells, promoting oxidative stress and reduced nitric oxide bioavailability.^24^ Aldosterone also increases the expression of adhesion molecules in both endothelial and coronary artery smooth muscle cells,^25^ fuelling inflammation through injurious leucocyte-endothelium crosstalk and leading to consequent extravasation.^26^

Patients with primary aldosteronism have structural and functional changes characteristic of diastolic dysfunction, with thickened ventricular walls, decreased mitral early peak velocity, and 20% lower peak myocardial early velocity.^27^ These mirror patients with heart failure and preserved ejection fraction, who experience high rates of postoperative morbidity and mortality after noncardiac surgery. From one database study involving 153 771 patients with preserved ejection fraction heart failure, 40% sustained cardiopulmonary morbidity, while non-cardiovascular complications including acute kidney injury and sepsis were recorded in 55% of patients.^28^ These complications were associated with a staggeringly high 5% in-hospital mortality rate and a 20% 30-day hospital readmission rate.

As a clinical consequence, high aldosterone levels are very likely associated with enhanced risk of cardiovascular events and mortality, especially when aldosterone secretion is inappropriate for renin levels and sodium intake. Cluster 3 also had markedly raised plasma ACE2 concentrations, which is associated with increased risk of major cardiovascular events.^29^ Higher plasma ACE2 concentrations have been reported in patients with primary aldosteronism.^30^ Patients in cluster 3 also demonstrated marked increases in arterial pressure on the day of surgery compared with their preoperative values. Comprising ∼10% of our study population, these data are consistent with a prevalence of resistant hypertension of 10–11% in the UK in apparently compliant patients aged 70 yr and older.^31^

In cluster 1, the combination of lower renin and elevated NT-proBNP levels was associated with similarly higher rates of myocardial injury to cluster 3. The significantly lower plasma renin levels in contrast to the higher levels displayed in the relatively cardioprotected cluster 2 are compatible with ineffective RAAS inhibition. ACEis and ARBs inhibit the formation and action of ANG-II, respectively, and consequently suppress the negative feedback inhibition of renin release by ANG-II.^32^ Accordingly, a compensatory increase in plasma renin concentration occurs accompanied by an increase in plasma renin activity.^33^ Inhibition of ACE also results in increased ANG-I levels, which can be converted to ANG-II by non-ACE-dependent pathways through reactive hyperreninaemia.^34^ Blockade of ANG-II stimulates renin synthesis and release indirectly through the action of ligands that activate the cAMP/PKA pathway in a Gsα-dependent fashion, including catecholamines, prostaglandins, and nitric oxide. The ACE inhibitors captopril and quinaprilate and the ARB candesartan increase plasma renin concentration 20- to 40-fold above basal levels in wild-type mice, but do not alter plasma renin concentrations in Gsα-deficient mice.^35^ Taken together, our data suggest that ineffective RAAS blockade over time failed to increase the plasma concentration of renin through the negative feedback effect of ANG-II on renin release. Moreover, our data suggest that interpretation of perioperative trials exploring the management of RAAS inhibitors should be refined by the use of relevant cardio-endocrine biomarkers such as NT-proBNP.

Strengths of our study are that all assays were batch analysed by investigators masked to clinical and trial data. Troponin thresholds used to define myocardial injury are consistent with international guidelines.^5^^,^^36^ A limitation was not establishing the aetiology of raised troponin by electrocardiographic criteria, although elevated troponin after surgery is associated with poorer outcomes regardless of aetiology.^4^ Routine preoperative echocardiography might have added further insights. Given the equal distribution of stopping or continuing RASis in each cluster, sampling before and after randomisation is likely inconsequential. The measurement of additional RAAS components, including Mas and Ang1-7, might also have added additional insights. Sampling before stopping the RASi might have been more instructive, but because of the logistic constraints of participants frequently being located far from referring hospitals, this was impractical. Given the magnitude of differences between the clusters, and the equal proportions of participants randomised to stop or continue RASi in each cluster, this does not appear likely to have been a major confounder in interpretation of the results.

In conclusion, preoperative renin–angiotensin–aldosterone system activation in patients undergoing noncardiac surgery is associated with myocardial injury. Exploring whether continuation of renin–angiotensin system inhibitors or preoperative intensification of renin–angiotensin–aldosterone system inhibition in these patients might help reduce perioperative myocardial injury is warranted.

## Authors’ contributions

Performance of ELISAs: AGDA

First and subsequent drafts of the manuscript: AGDA, TEFA, SB, PD, GLA

Trial statistics: AP

First draft of the manuscript: DB, RMP

Study design, interpretation of results: VK

Study conception, design, analysis: GLA

## Funding

The UK National Institute for Academic Anaesthesia (British Oxygen Company research chair grant to GLA); NIHR Advanced Fellowship (NIHR300097), and 10.13039/501100000274British Heart Foundation Programme grants (RG/14/4/30736; RG/19/5/34463). TEFA and RMP were supported by NIHR.

## Declarations of interest

GLA, RMP, DB, TEFA receive support from NIHR (National Institute for Health and Care Research). GLA and RMP are editors of the *British Journal of Anaesthesia*, and TEFA is the social media editor. The remaining authors have no disclosures to report.
