# Supplementary material for: Preoperative activation of the renin–angiotensin system and myocardial injury in noncardiac surgery: exploratory mechanistic analysis of the SPACE randomised controlled trial
Source: Br J Anaesth. 2024 Dec 20;134(5):1300–7. doi: 10.1016/j.bja.2024.10.040 (PMC12106892; doi:10.1016/j.bja.2024.10.040)

**Activation of the Renin–Angiotensin system and myocardial injury in noncardiac surgery: Post Hoc Analysis of the SPACE randomised controlled Trial**

Ana Gutierrez del Arroyo PhD,^1^ Tom. E. F. Abbott PhD, ^1^ Akshaykumar Patel PhD, ^1^ Salma Begum, ^1^ Priyanthi Dias PhD,^1^ Sameer Somanath FRCA,^2^ Alexander Middleditch FRCA,^3^ Stuart Cleland FRCA,^4^ David Brealey PhD,^5^ Rupert M. Pearse MD,^1^ Vikas Kapil PhD,^6^ Gareth L. Ackland PhD ^1^

SUPPLEMENTARY DATA

Contents

[R code and results- NBClust 2](#_Toc179732291)

[R data plot 3](#_Toc179732292)

[Sensitivity analysis excluding beta-blockers- primary outcome. 4](#_Toc179732293)

[ELISA standard curves for this study 4](#_Toc179732294)

[Sensitivity analysis excluding beta-blockers- plasma RAAS components. 5](#_Toc179732295)

[Post hoc multivariable analysis 6](#_Toc179732296)

# R code and results- NBClust

To find the optimal number of clusters in our population, the NbClust package was used Charrad M et al. It provides 30 indices which determine the number of clusters in a dataset, and it also offers the best clustering scheme from different results to the user. This enables the user to simultaneously evaluate several clustering schemes while varying the number of clusters, to help in determining the most appropriate number of clusters for the dataset of interest. The distance measures available in the NbClust package are: Euclidean distance, maximum distance, Manhattan distance, Canberra distance, binary distance and Minkowski distance. Several agglomeration methods are also provided by the NbClust package- Ward, single, complete, average, McQuitty, median and centroid.

res <-NbClust(data, distance = "euclidean", min.nc = 2, max.nc = 8, method = "ward.D2",

+ index = "duda")

>res$All.index

| **Cluster n=** | **2** | **3** | **4** | **5** | **6** | **7** | **8** |
| --- | --- | --- | --- | --- | --- | --- | --- |
| Value index | 0.5751 | 1.8076 | 0.7068 | 0.9234 | 0.2364 | 0.6123 | 0.6246 |

> res$Best.nc

Number_clusters Value_Index

3.0000 1.8076

> res$Best.partition

SPACE patient ID at start of each row denoted in square brackets [patient ID]

[1] 1 1 1 1 1 1 1 1 1 1 1 1 1 1 2 1 2 1 **3** **3** **3** **3** **3** **3** **3** **3** **3** **3** 2 **3** 2 **3** **3** **3** 2 **3** **3** **3**

[39] **3** **3** **3** **3** **3** **3** **3** **3** **3** **3** **3** 2 **3** **3** **3** **3** **3** **3** **3** **3** **3** **3** **3** **3** **3** **3** **3** **3** **3** **3** **3** **3** **3** **3** **3** **3** **3** **3**

[77] **3** **3** **3** **3** **3** **3** **3** **3** **3** **3** **3** **3** **3** **3** **3** **3** **3** **3** **3** **3** **3** 2 **3** **3** **3** **3** **3** **3** 2 **3** **3** **3** **3** **3** **3** **3** **3** **3**

[115] **3** **3** **3** **3** **3** **3** **3** 2 **3** **3** **3** 2 **3** **3** **3** 2 **3** **3** **3** **3** **3** **3** **3** **3** **3** **3** **3** **3** **3** **3** **3** **3** **3** **3** **3** **3** **3** **3**

[153] **3** **3** **3** **3** **3** **3** **3** **3** **3** **3** **3** **3** **3** **3** **3** **3** 2 **3** **3** **3** **3** **3** **3** **3** **3** **3** **3** **3** **3** **3** 2 **3** **3** **3** **3** **3** **3** **3**

[191] **3** **3** **3** **3** **3** **3** **3** **3** **3** **3** **3** **3** **3** **3** **3** **3** **3** **3** **3** **3** **3** 2 **3** **3** **3** **3** **3** 2 **3** **3** **3** **3** **3** **3** **3** **3** **3** **3**

[229] **3** **3** **3** **3** **3** **3** **3** 2 **3** **3** **3**

#
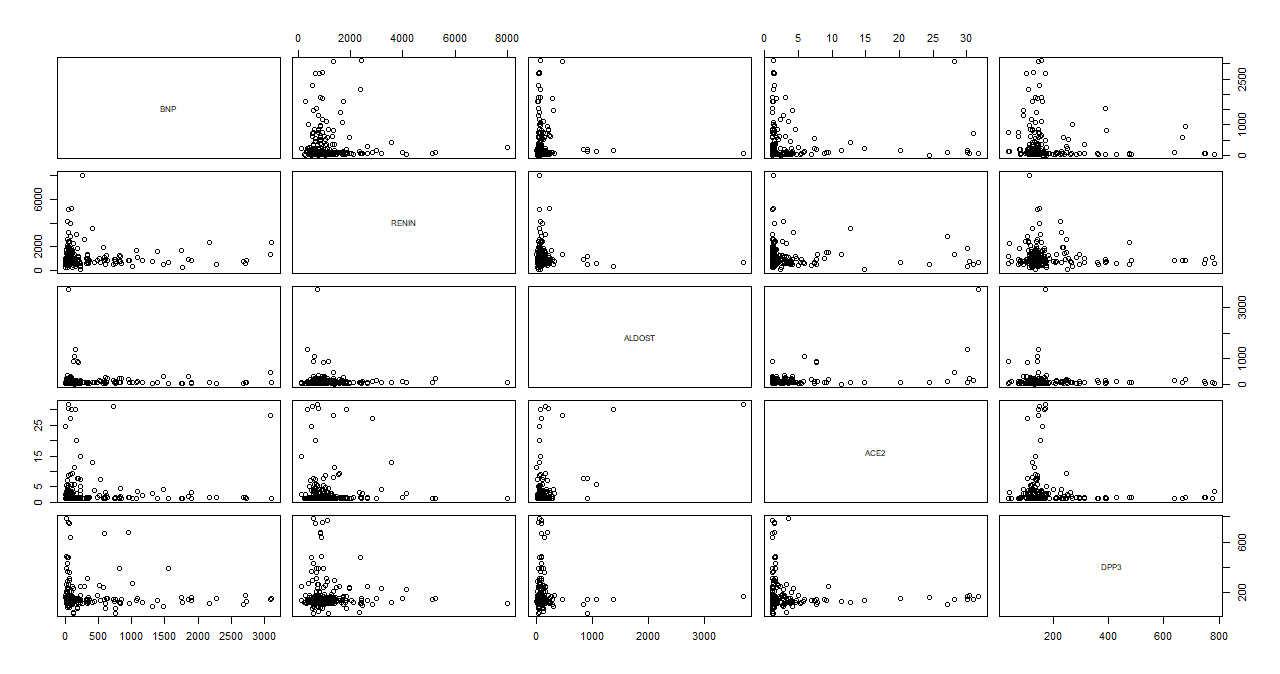
R data plot

# Sensitivity analysis excluding beta-blockers- primary outcome.

|  | Myocardial injury | Total | % myocardial injury |
| --- | --- | --- | --- |
| Cluster 1 | 14 | 31 | 45.2% |
| Cluster 2 | 35 | 122 | 28.7% |
| Cluster 3 | 7 | 15 | 46.7% |

# ELISA standard curves for this study

All y-axes denote protein standard values.


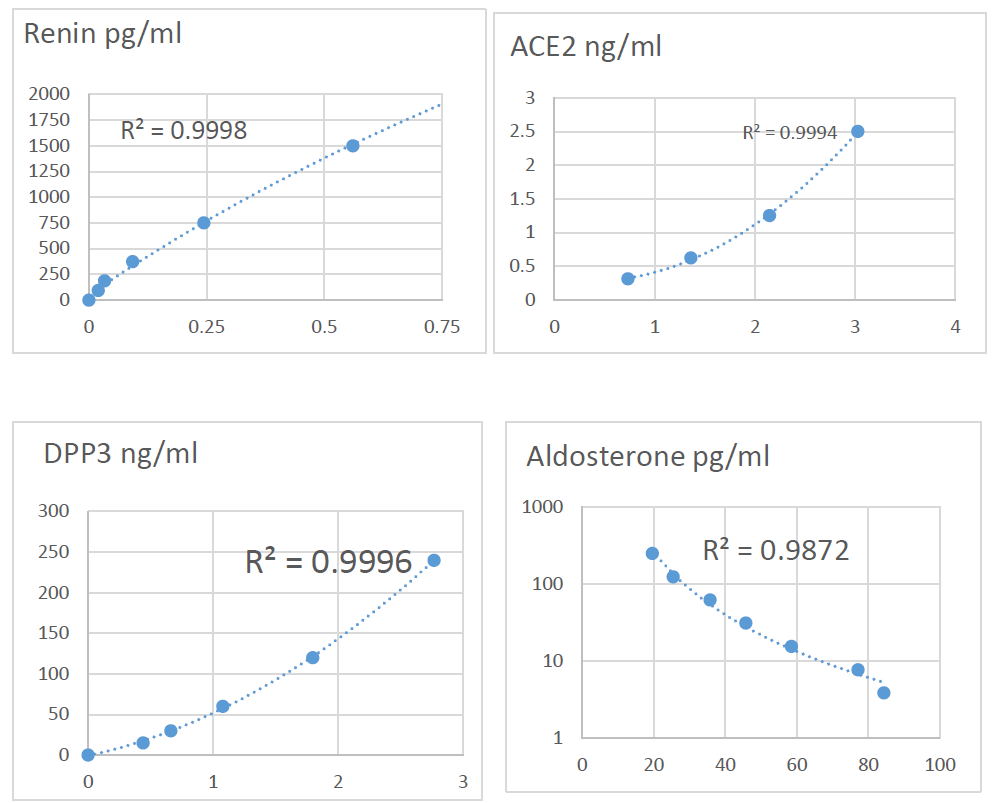


# Sensitivity analysis excluding beta-blockers- plasma RAAS components.

Preoperative plasma values for NT-proBNP, renin, aldosterone, ACE2 in each cluster, without patients on beta-blockers. P values refer to main difference found in whole dataset.


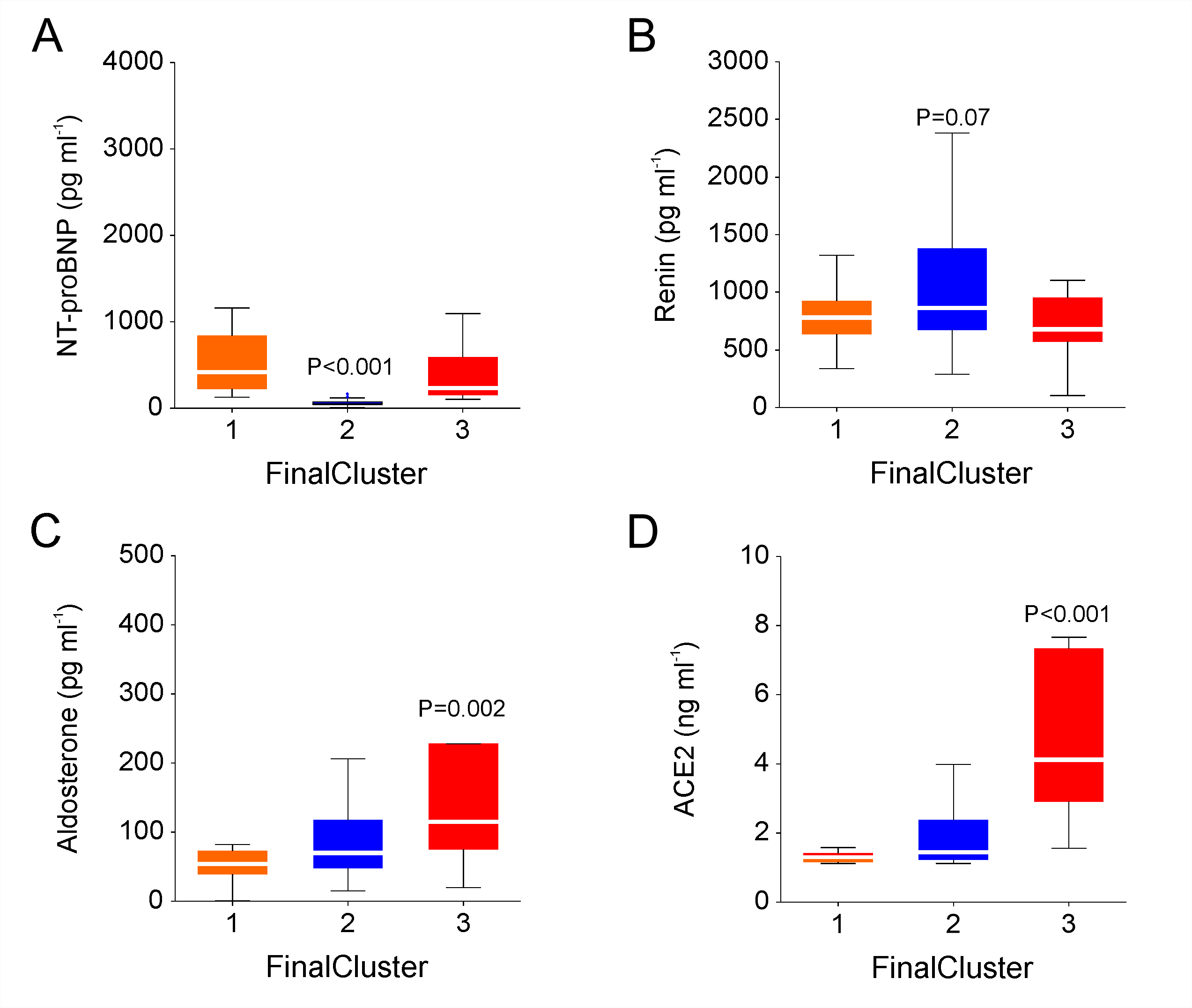


# Post hoc multivariable analysis

BNPtype=1 is >100ng/ml; gender_core=2: male;


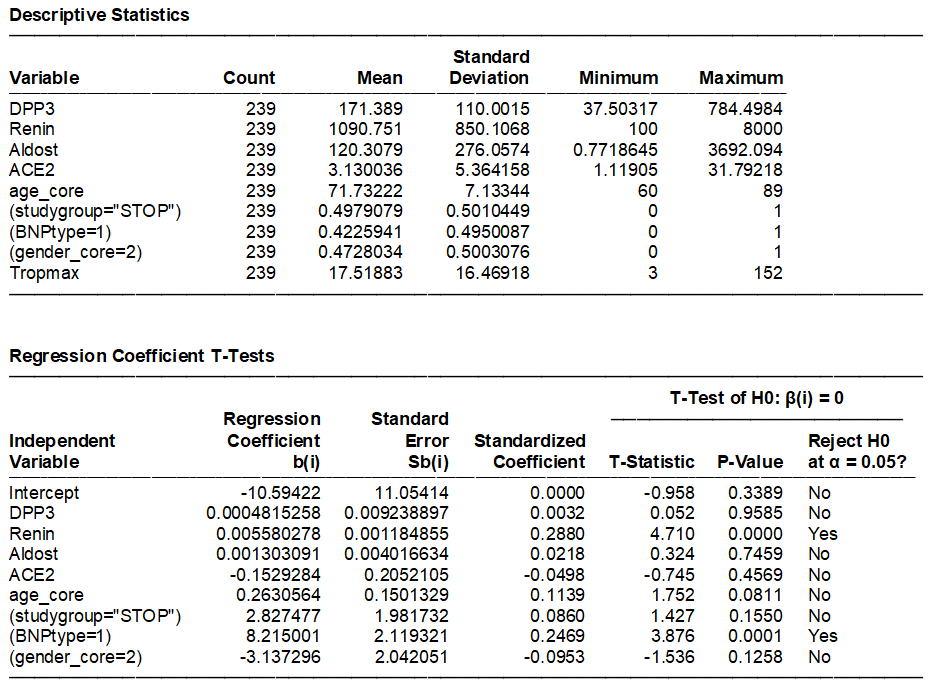

Supplement: Multimedia component 1 [file mmc1.docx]
